# Case Report: Idiopathic Subcutaneous Thrombotic Vasculopathy

**DOI:** 10.3389/fmed.2022.843793

**Published:** 2022-04-15

**Authors:** Kerilyn Godbe, Ashlie Elver, Peter Chow, Chris Williams, Garth Fraga, Penelope Harris, Mohammed Taha, Dhaval Bhavsar, Richard Korentager

**Affiliations:** ^1^Department of Plastic Surgery, University of Kansas Medical Center, Kansas City, KS, United States; ^2^University of Kansas School of Medicine, Kansas City, KS, United States; ^3^Department of Dermatology, University of Kansas Medical Center, Kansas City, KS, United States; ^4^Department of Internal Medicine, University of Kansas Medical Center, Kansas City, KS, United States; ^5^Department of Pathology, University of Kansas Medical Center, Kansas City, KS, United States

**Keywords:** vasculopathy, calciphylaxis, ulcers, COVID, polyarteritis nodosa, thrombophilia

## Abstract

Lower extremity ulcers have significant morbidity, with treatment determined by the underlying disorder. Reported is a 32-year-old female presenting with small skin nodules and bruises across her legs 4 weeks following her second COVID vaccination. These lesions progressed into large, necrotic ulcers over several months. Initial work-up showed widespread pannicular thrombotic vasculopathy with ischemic skin necrosis. The tissue was negative for calcification on Von Kossa histochemistry, and a working diagnosis of subcutaneous thrombotic vasculopathy was suggested. The ulcers progressed despite treatments with corticosteroids, therapeutic anticoagulation, intravenous immunoglobulin, plasmapheresis, sodium thiosulfate, wound care, and repeat debridement. Later debridement specimens demonstrated rare vascular and pannicular calcifications. This finding supports the hypothesis that subcutaneous thrombotic vasculopathy is a precursor to calciphylaxis, the patient’s current working diagnosis. However, based on the patient’s entire clinical picture, a definitive diagnosis has yet to be found. This report highlights the challenges of working with rare diseases and the importance of multidisciplinary cooperation.

## Introduction

Chronic lower extremity ulcers affect over 7 million patients in the United States per year (1). Common causes of lower extremity ulcers include venous insufficiency, arterial insufficiency (ischemia), and neuropathy. Less frequent causes of ulcers include infection, neoplasms, hypercoagulable states, pyoderma gangrenosum, and hematologic disease. Ulcers with a presentation of retiform purpura or livedo racemosa are rare and suggest either systemic vasculitis or occlusive vasculopathy (1). Calciphylaxis, a rare yet highly morbid type of occlusive vasculopathy, is characterized by extensively necrotic and painful ulcers on fatty areas such as the abdomen, buttocks, and thighs (1, 2).

Thrombosis secondary to SARS-CoV2 (COVID-19) infection is well recognized. Thrombosis following vaccination against COVID-19 is very rare (3, 4). To our knowledge, this is the first case of post-COVID vaccination thrombotic vasculopathy resulting in progressive necrotic ulcers. Here, we describe a case of calciphylaxis-like occlusive thrombotic vasculopathy which commenced four weeks after the second dose of mRNA COVID-19 vaccination. We discuss the differential with other ulcerating skin diseases with vascular pathology, including vasculitis, subcutaneous thrombotic vasculopathy, and non-uremic calciphylaxis.

## Case Description

**FIGURE 1:**
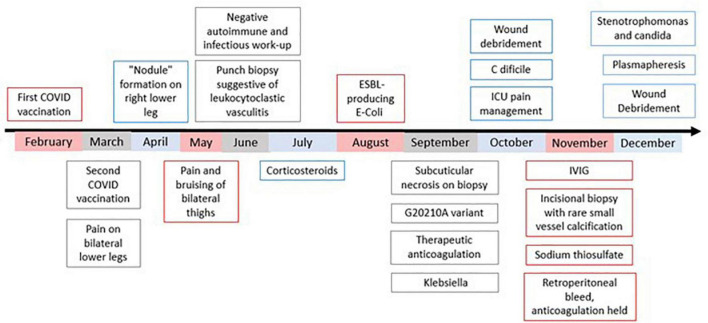
Chronological depiction from patient’s initial presentation to current working diagnosis. Work-up prior to September was performed at outside institutions. In September, the patient was admitted to our institution for treatment. At no point during this course have lesions stopped progressing. In early February, the patient unfortunately passed away.

A 32-year-old female presented to her primary care physician with tender erythematous nodules on her bilateral lower extremities four weeks following her second Moderna COVID vaccination (Figure 1). She had a past medical history of Ehlers-Danlos syndrome, psoriasis, 3 possible first-trimester pregnancy losses, and chronic diarrhea. There were no other family members with similar conditions. Serologic testing for SS-A, SS-B, JO-1, RF, CCP, ANA, DNAse B, TG2, PR3/MPO with reflex to ANCA, protein electrophoresis with immunofixation, complement C3c and C4c, tuberculosis, and hepatitis B and C was negative. A punch biopsy was reported by a community pathologist to be consistent with leukocytoclastic vasculitis. Additional testing for HIV antigen/antibody and Lyme disease was negative. Vascular studies revealed no abnormalities. As her clinical presentation did not match a small vessel vasculitis, a provisional diagnosis of cutaneous polyarteritis nodosa (PAN) was made by a community rheumatologist in the setting of ulcerating retiform purpura consistent with a medium vessel process.

Treatment with 50 mg oral prednisone daily, 0.6 mg colchicine twice daily, and daily wound care with Medihoney for suspected cutaneous PAN was ineffective. Her dose of prednisone was decreased to 40 mg. One week later, the patient reported “a feeling of gravel” on her legs. At this time, her legs contained many necrotic ulcers and nodules, requiring debridement under anesthesia at an outside institution. Cultures showed extended spectrum beta-lactamase (ESBL)-producing *E. coli* and she was started on intravenous (IV) vancomycin and fluconazole, with collagenase and xeroform wound care.

In early September, the patient was admitted to our institution for pain control and wound management, with her prior antibiotic regimen stopping 3 days prior. Estrogen based hormones were stopped at this time, and she was started on ertapenem 1 g IV and fluconazole 400 mg daily with wounds dressed in sulfamylon cream. Alternative diagnoses were discussed at this time due to lack of response to treatment of medium vessel vasculitis, including pyoderma gangrenosum. Repeat autoimmune and infectious testing was negative. A colonoscopy showed no evidence of inflammatory bowel disease. Magnetic resonance imaging (MRI) of her chest, abdomen, and pelvis was negative for vasculitis. The initial outside punch biopsy was reviewed, and the pathology findings were reclassified as thrombotic vasculopathy instead of vasculitis. Repeat punch biopsy revealed subcuticular necrosis with fibrin thrombi within blood vessels. No significant immunoglobulin (Ig) G, IgA, IgM, or C3 deposits were identified *via* direct immunofluorescence. Testing for coagulopathies was positive for prothrombin variant G20210A. The remainder of her thrombophilia evaluation, including testing for cold agglutinins, JAK2 with reflex, paroxysmal nocturnal hemoglobinuria, antithrombin III deficiency, factor V Leiden, platelet factor 4 antibodies, antiphospholipid labs, platelet count, and protein C and S levels were normal. The patient started taking warfarin due to concern of vasculopathy and was transitioned to apixaban once negative antiphospholipid antibodies were confirmed. Her antibiotic regimen was altered to 3 g IV ampicillin-sulbactam and 100 mg micafungin daily after a wound swab grew *Klebsiella*.

**FIGURE 2:**
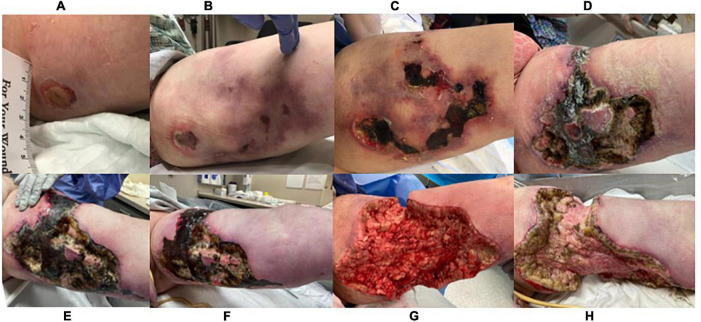
Lesion progression of left medial thigh over 3.5 months. **(A)** 8/27/21**, (B)** 9/11/21, **(C)** 9/27/21, **(D)** 10/26/21, **(E)** 11/16/21, **(F)** 11/30/21, **(G)** 12/1/21, taken immediately post debridement in operating room **(H)** 12/7/21.

In October, a diagnosis of cutaneous PAN was no longer favored due to evidence of ulcers eroding further into adipose tissue despite patient’s steroid treatment (Figure 2). The patient’s steroid treatment was tapered. After being on prolonged antibiotic treatments, the patient developed diarrhea secondary to clostridium difficile infection. Oral vancomycin was added, and she underwent two surgical debridements to lessen the load of necrotic tissue. Her anticoagulant regimen was switched to a heparin drip prior to the first procedure. By the end of October, it was discovered the patient was resistant to heparin. At the end of this month, the patient was transferred to the intensive care unit for sedation due to uncontrollable pain.

**FIGURE 3:**
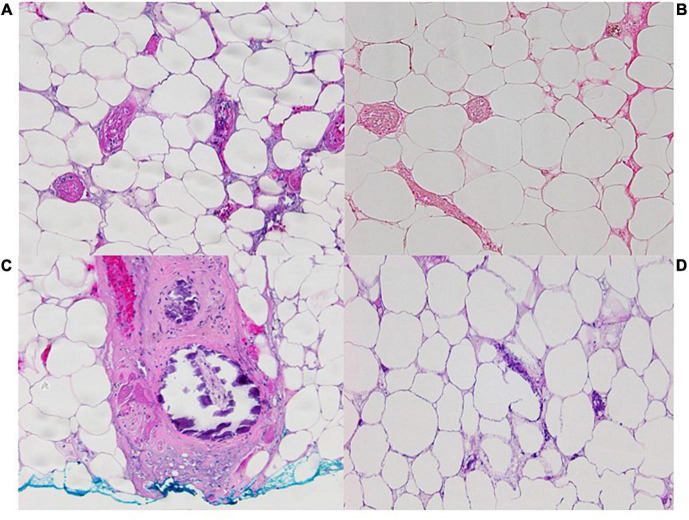
Pathology images from incisional biopsy on left flank on 11/12/21 at 100x OM with **(A)**. Widespread pannicular thrombotic vasculopathy with ischemic pannicular necrosis (H&E) **(B)**. Negative for calcification on Von Kossa histochemistry with **(C)**. A single subcutaneous arteriole with mural calcification at the biopsy base (H&E). **(D)** Pathology image from incisional biopsy 12/1/21 at 100x OM with H&E showing adipocyte calcification.

By the beginning of November, the patient received her third debridement. The most likely cause of the patient’s progressive necrotic ulcers at this time was thought to be a thrombotic vasculopathy. A review of the literature found a case series on subcutaneous thrombotic vasculopathy similar to the patient’s presentation. The possibility of an autoimmune reaction following COVID vaccination was discussed by the hematology and immunology teams at this time. A larger incisional biopsy at the edge of a developing lesion on her flank was performed. She was started on therapeutic enoxaparin, 81 mg aspirin daily, dipyridamole 75 mg four times daily, and pentoxifylline 400 three times a day in addition to treatments with IV immunoglobulins. Despite appropriate 1 mg/kg twice daily dosing of enoxaparin, the patient’s anti-factor X activity level was subtherapeutic requiring escalation to 1.5 mg/kg twice a day. In addition, the patient was given 1 g IV solumedrol and transitioned to prednisone. The biopsy results came back as diffuse small vessel thrombosis with rare small vessel calcification, suggesting a thrombotic vasculopathy (Figure 3). Despite the less conspicuous tissue calcification present in the patient’s biopsy, calciphylaxis was considered as a diagnosis. The low risk of IV sodium thiosulfate was weighed with the potential benefits, and it was started twice weekly. Over the next 2 weeks, the patient experienced improvement in pain – however during this time she started oral ketamine and received a hydromorphone PCA.

**FIGURE 4:**
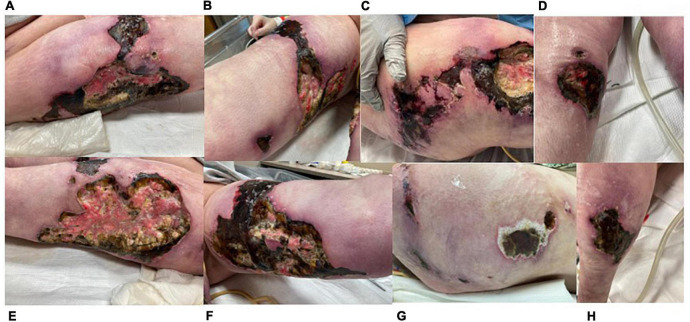
Images of progressive necrotic ulcers taken 11/30/21 (other than C). **(A)** Right lateral thigh. **(B)** Right medial thigh. **(C)** Right flank (11/23/21). **(D)** Right anterior lower leg. **(E)** Left lateral thigh. **(F)** Left medial thigh. **(G)** Left flank. **(H)** Left posterior lower leg. Lesions not shown: right posterior lower leg, left anterior lower leg.

In mid-November, the patient had a spontaneous retroperitoneal bleed resulting in discontinuation of therapeutic anticoagulation. By the end of November, pentoxifylline was restarted with prophylactic enoxaparin. Wounds continued to progress (Figure 4), most notably on patient’s bilateral flanks. The decision was made to stop intravenous immunoglobulin and initiate plasmapheresis in attempt to reverse any unknown immune-mediated processes. Her bilateral legs were debrided again early December 2021 to decrease necrotic tissue burden. She is now on argatroban due to difficulty with enoxaparin dosing, has received 7 treatments of plasmapheresis, and is receiving IV sodium thiosulfate 25 g five times weekly. A recent tissue culture was positive for *Stenotrophomonas maltophilia*, *Mucor*, and *Candida parapsilosis*, and her antibiotic regimen consists of 100 mg minocycline twice daily, 1 g imipenem/cilastin intravenous every 8 h, 5 mg/kg IV amphotericin B every 24 h as tolerated, and oral 125 mg vancomycin four times daily.

Her prognosis remained guarded, with differential diagnoses including idiopathic subcutaneous thrombotic vasculopathy, non-uremic calciphylaxis sin calcifications, or an autoimmune response to her COVID vaccination. She reported depression, and high levels of pain despite a rigorous pain control regimen. She chose not to see the lesions on her bilateral lowerextremities since November as she believed it would worsen her mental health. Although it was difficult at times to havehope, she and her family continued to search for answers and have aggressive treatment goals. She wanted to share her story with the medical field in hope other providers would be able to provide insight to help her, and future patients with similar afflictions. In early February, the patient was transitioned to comfort care and passed away surrounded by her family.

## Discussion

We report a case of idiopathic subcutaneous thrombotic vasculopathy four weeks following the second dose of mRNA COVID vaccination in a patient with Ehlers Danlos syndrome. It is unknown whether subcutaneous thrombotic vasculopathy is a unique disease, a manifestation of calciphylaxis, or other thrombophilia. For this patient, other possible causes of thrombophilia include an inherited thrombophilia or novel form of vaccine induced thrombophilia.

Zembowicz and co-workers coined the term subcutaneous thrombotic vasculopathy syndrome to describe three patients with diffuse subcutaneous capillary and arteriolar thrombosis associated with ischemic skin necrosis. The findings were similar to those seen in calciphylaxis, but devoid of tissue calcifications (5). The histopathology is identical to that in our patient’s initial biopsies. Unlike our case, the patients described by Zembowicz et al. were older with serious underlying medical conditions. Zembowicz et al. also found that 73% of cases of conventional calciphylaxis had foci of subcutaneous thrombi without calcifications and raised the possibility that subcutaneous thrombotic vasculopathy may represent calciphylaxis *sine* calcifications (5).

Calciphylaxis is a highly morbid and often fatal vasculopathy usually associated with end stage renal disease (ESRD). Early clinical findings of this disease include erythema, induration, and severe pain out of proportion to the physical exam (6). With time, papules and plaques coalesce into retiform purpura, which will progress to eschar and ulcer formation (6). These exam findings are thought to be secondary to medial layer calcification of the vessels in the subcuticular adipose layer, followed by subintimal fibrosis and thrombus formation (2). There are rare reports of non-uremic calciphylaxis in patients without prior renal impairment. Risk factors in patients without ESRD include female sex, obesity, hypercoagulability, autoimmune disorders, other connective tissue disorders, and medications such as Warfarin and corticosteroids (5, 7, 8). All such risk factors are present in this patient. For patients with end stage renal disease matching the clinical presentation of calciphylaxis, a biopsy is not necessary to make the diagnosis (6). In the absence of ESRD, biopsy is recommended.

McMullen et al. found a sensitivity of 0.85 and specificity of 0.88 for von Kossa histochemistry for diagnosis of calciphylaxis (9). Von Kossa staining was negative for tissue calcifications in our patient’s initial punch biopsy specimens. However, the clinical efficacy of punch biopsies can be low if the quantity or depth of tissue obtained is not enough for diagnosis, a limitation of this patient’s initial testing (10). Deep incisional cutaneous biopsy however provides adequate tissue for histologic study (11) as shown whensubsequent larger tissue samples revealed focal sparse subcutaneous vascular and adipocyte calcifications visible on routine hematoxylin-eosin- stained histologic sections. While the calcification is not as diffuse as classical calciphylaxis, these later biopsy specimens support the hypothesis that subcutaneous thrombotic vasculopathy syndrome is a precursor to calciphylaxis. However, this patient still does not have the traditional risk factors for calciphylaxis, did not manifest calcium, phosphorus, or parathyroid hormone elevation, and failed to respond to therapeutic anticoagulation and sodium thiosulfate.

Physicians should be aware of atypical presentations of calciphylaxis as treatments for common differentials, such as vasculitis and vasculopathies, can worsen the disease. The patient described was originally treated with corticosteroids for suspected cutaneous PAN, in addition to Warfarin for suspected vasculopathy. These treatment regimens were decided based on the current literature and are not unique to this patient alone. Notably, 60–80% of patients with non-uremic calciphylaxis were treated with corticosteroids prior to the development of calciphylaxis and 25–60% of patients had a history of warfarin administration (12, 13).

There are elements in this patient’s history suspicious for inherited thrombophilia. She reported three pregnancy losses in the first trimester. Pregnancy loss in the first trimester is more consistent with antiphospholipid syndrome, but testing was negative for antiphospholipid antibodies (14). Late fetal losses and family and personal history of thromboemboli are suggestive of inherited thrombophilia (14). This patient has a prothrombin mutation, but not the classic disease history. Her resistance to heparin is notable. Heparin resistance could be due to non-specific binding, antithrombin deficiency, platelet interactions, elevated coagulation factors, adexanet alfa administration, or infection with COVID-19 (15). This patient’s thrombophilia work-up, and lack of adexanet alfa administration rule out all causes except non-specific binding, which can cause a wide variability in patient response and dose requirement (15). This variability could explain the increased heparin requirements followed by retroperitoneal bleed. Ultimately, there is some level of inherited thrombophilia indicating need for indefinite anticoagulation in the future which could be contributing, if not causing, her disease process.

One last important feature of this case is the timing of the patient’s COVID-19 vaccination prior to disease onset. Multiple reports have discussed concerns regarding thromboembolic events, macro and microvascular, related to the COVID disease and vaccine (3, 16, 17). Magro et al. examined skin, lung, and various other tissues in patients with mild to fatal COVID-19. In their Weill Cornell and Regional Medical Laboratory review from March of 2020 to June of 2021, 14 cases of patients with moderate to severe COVID were found to have cutaneous lesions attributable to complement-mediated microvascular injury due to spike glycoprotein activation of the complement pathway and a procoagulant state (17–19). This same group also identified 13 cases of cutaneous reactions developing 1 day to 7 weeks following COVID-19 vaccination (9 Moderna, 2 Pfizer, remainder vaccine type unknown). The most common cutaneous skin manifestation was noted to be eczematous dermatitis, but other cutaneous findings of patients included urticarial vasculitis, Grover disease, Herpes Zoster, and perniosis (17). Most of these cases were determined to be a self limited type IV delayed hypersensitivity reaction. The cases of post vaccine vasculitis were believed to be humorally mediated however, consisting of a type III immune complex reaction of the spike glycoprotein bound to antibody (17). Upon tissue sample analysis of the post-vaccination patients, microvascular localization of spike glycoproteins was observed *via* viral spike glycoprotein immunohistochemistry (IHC). Interestingly, this finding is similar to the findings of patients with severe/critical COVID19 with thrombotic retiform purpura (17).

This information is interesting in the context of this case as the patient discussed presented with cutaneous symptoms following her COVID19 vaccination. Although similar cutaneous symptoms have been identified in severe cases of patients with COVID19, they have not been observed following vaccination despite the similarity of microvascular localization of the spike protein. In contrast to the post vaccination patients in the review by Magro et al. the patient in this case did not have spike glycoprotein IHC performed, and her skin lesions did not resolve either spontaneously or with systemic steroid treatment. Additionally, her complement levels were within normal limits and she had negative direct immunofluorescence with no significant IgG, IgA, IgM, or C3 deposits identified on pathology specimens. However, given our evolving information about the COVID vaccination, it is possible that this patient’s vaccination may have been one of many factors contributing to her unique presentation.

This case report is limited by lack of a definitive diagnosis and effective treatment. This complex case is important to the literature in order to spread awareness of atypical presentations of calciphylaxis and prevent physicians from doing accidental harm by prescribing regimens that worsen the disorder, such as warfarin and corticosteroids. Without a specific diagnosis or effective method of preventing disease progression, the patient passed away in early February of 2022. It is our hope that further discussion of this patient’s case will help similar patients in the future.

## Data Availability Statement

The original contributions presented in the study are included in the article/supplementary material, further inquiries can be directed to the corresponding author.

## Ethics Statement

Written informed consent was obtained from the individual(s) for the publication of any potentially identifiable images or data included in this article.

## Author Contributions

KG wrote the case description, portions of the discussion section, created figures, and cared for the patient. AE wrote portions of the discussion section. GF assisted with pathology interpretation, literature review, and thorough editing of manuscript. PC, CW, PH, MT, DB, and RK assisted with editing the manuscript, in addition to caring for the patient. All authors agreed to be accountable for the content of this work.

## Conflict of Interest

The authors declare that the research was conducted in the absence of any commercial or financial relationships that could be construed as a potential conflict of interest.

## Publisher’s Note

All claims expressed in this article are solely those of the authors and do not necessarily represent those of their affiliated organizations, or those of the publisher, the editors and the reviewers. Any product that may be evaluated in this article, or claim that may be made by its manufacturer, is not guaranteed or endorsed by the publisher.
